# Editorial: Innovative vaccine strategies for enhanced flavivirus immunization

**DOI:** 10.3389/fcimb.2025.1729523

**Published:** 2025-11-19

**Authors:** Srinivasa Reddy Bonam, Nitin K. Saksena, Pedro A. Reche

**Affiliations:** 1 Vaccine Immunology Laboratory, Department of Applied Biology, CSIR-Indian Institute of Chemical Technology, Hyderabad, India; 2 Academy of Scientific and Innovative Research, Ghaziabad, India; 3 ARNA Life Sciences & ARNA Pharma, Sydney, NSW, Australia; 4 Department of Immunology, School of Medicine, Complutense University of Madrid, Madrid, Spain

**Keywords:** flavivirus, virus-host interaction, innate immune response, adaptive immune responses, vaccines

## Introduction

**Figure 1:**
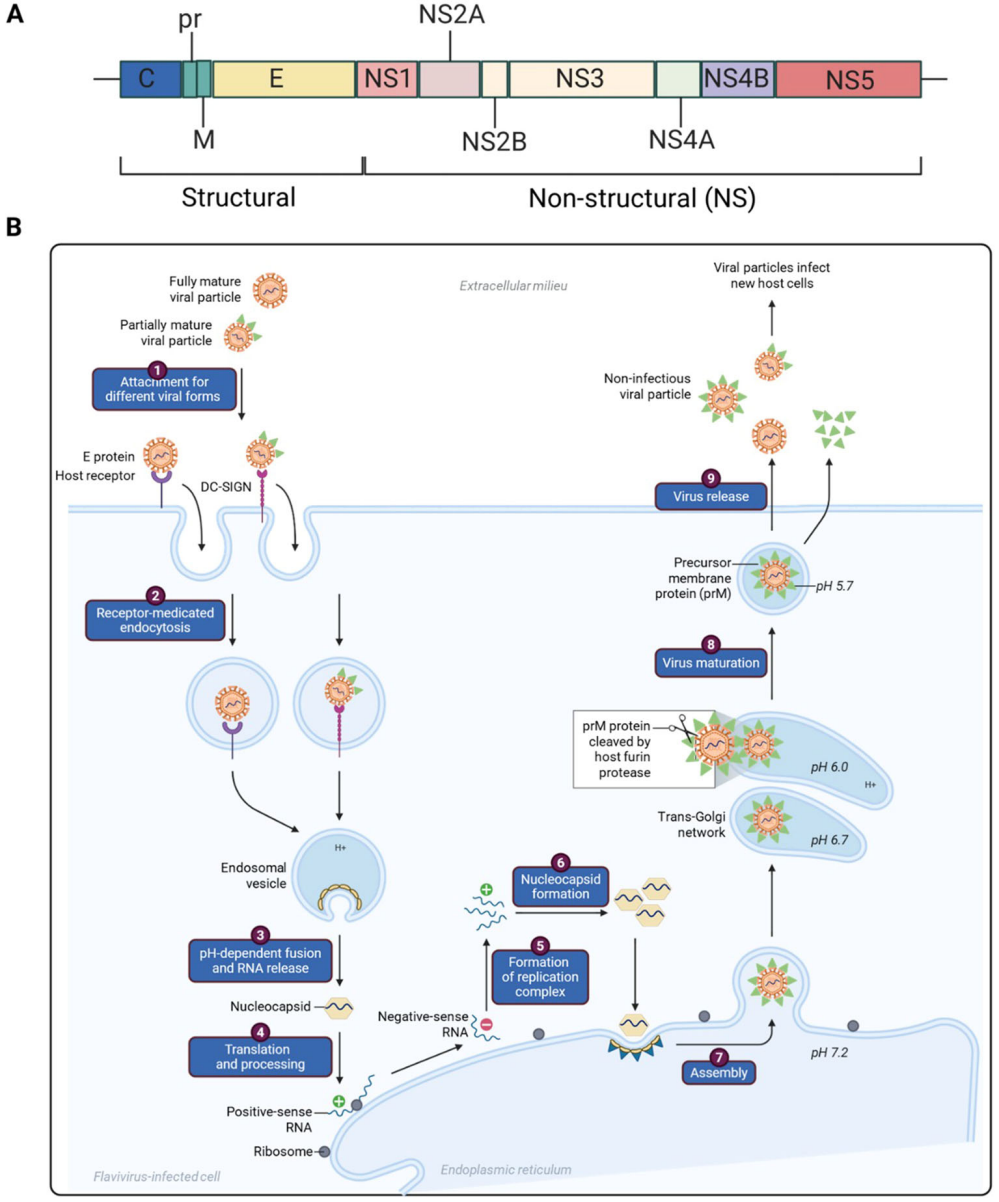
Generic flaviviral genome organization and life cycle. **(A)** The viral genome consists of a single open reading frame (ORF) flanked by 5′ and 3′ UTRs. This genomic structure is highly conserved across the Flavivirus genus (Pierson and Diamond, 2020). Once inside the host cell, the ORF is translated by host ribosomes into a large polyprotein. This polyprotein is then cleaved by both host and viral proteases into three structural proteins (C, prM, and E) and seven non-structural (NS) proteins: NS1, NS2A, NS2B, NS3, NS4A, NS4B, and NS5 (Pierson and Diamond, 2020). **(B)** Flaviviruses gain entry into mammalian cells by engaging a variety of host attachment molecules, including those that recognize the viral envelope or N-linked glycans present on the virion surface (Gould and Solomon, 2008). Among these, C-type lectin receptors on the cell membrane not only facilitate viral binding but can also trigger intracellular signaling pathways that influence immune responses. Once attached, the virus is taken up *via* clathrin-mediated endocytosis (Pierson and Diamond, 2020), hijacking cellular machinery typically used to internalize large biomolecules. Inside the host cell, the virus fuses with endosomal membranes in a process activated by acidic pH (Mukhopadhyay et al., 2005), allowing release of the viral genome. Subsequent replication of viral RNA occurs on specialized membrane structures that are Remodeled by viral nonstructural proteins (Neufeldt et al., 2018), these NS proteins play diverse and essential roles in viral replication and progeny production. Among them, NS3 functions as a helicase and, when paired with NS2B (forming the NS2B3 complex), acts as the viral protease. NS5 has two enzymatic activities: it functions as the RNA-dependent RNA polymerase and as a methyltransferase, enabling the synthesis and capping of new viral RNA genomes. Viral genome replication occurs within specialized structures derived from the remodeled endoplasmic reticulum (ER), known as replication organelles or compartments. These compartments concentrate the necessary replication components and shield viral RNA from host immune detection. Inside these compartments, NS proteins assemble into a replication complex that drives RNA synthesis. Initially, the positive-sense single-stranded RNA (ssRNA^+^) serves as a template to produce negative-sense RNA (ssRNA^−^), which is then used to generate additional ssRNA^+^ genomes (Slon Campos et al., 2018; Stiasny et al., 2023; Madere et al., 2025). These new genomes are either further amplified or packaged into new virions. Beyond their role in RNA synthesis, NS proteins also contribute to ER remodeling and help modulate the host immune response, ensuring efficient viral replication and evasion of host defenses (Slon Campos et al., 2018; Pierson and Diamond, 2020).

Flaviviruses, arthropod-transmitted viruses (arboviruses) within the family *Flaviviridae*, positive-sense, single-stranded (ssRNA+), cause major diseases including yellow fever (YFV), dengue (DENV), Zika (ZIKV), chikungunya (CHIKV), Japanese encephalitis (JEV), Tambusu virus (TMUV), Tick-borne encephalitis virus (TBEV), West Nile fever (WNV), and others contributing to several million infections annually (Gould and Solomon, 2008). Flavivirus has a full-length genome of about 11 kb. The generic flaviviral genome organization and life cycle are depicted in **Figure 1**. The structural organization of flaviviruses and host-pathogen interactions has been excellently presented in several outstanding reviews (Mukhopadhyay et al., 2005; Neufeldt et al., 2018; Pierson and Diamond, 2020; Stiasny et al., 2023; Madere et al., 2025). Vaccination is the most effective preventive strategy, yet current vaccines face challenges in efficacy, safety, and coverage. Recent innovations, such as nucleic acid-based platforms, structural vaccinology, and genetically engineered microorganisms, offer promise. Still, critical gaps remain in understanding host-virus dynamics and refining vaccine delivery and production.

The ongoing global challenge of Flavivirus infections—including ZIKV, DENV, and WNV—highlights the urgent need for continual advancements in vaccine development. This Research Topic, “Innovative Vaccine Strategies for Enhanced Flavivirus Immunization”, presents a collection of research that charts a path forward by emphasizing improved safety, effectiveness, and cross-protection. The articles unify around a common goal: moving beyond traditional methods to create precise, durable, and safer immune defenses.

This Research Topic centers on four key strategic shifts: employing precision and rational design for antigens and delivery systems; explicitly addressing immunopathology such as Antibody-Dependent Enhancement (ADE); emphasizing robust cellular (T cell) immunity alongside humoral responses; and leveraging advanced technologies like immunoinformatics and sophisticated delivery platforms.

## Understanding host-virus interactions: the cellular battle

Despite robust adaptive immune responses, how flavivirus evasion tactics enable persistence, transmission, and immunopathology have been elucidated elsewhere (Slon Campos et al., 2018; Pierson and Diamond, 2020). MicroRNAs (miRNAs), small non-coding RNAs of 20–24 nucleotides, are key post-transcriptional regulators that influence gene expression by binding to the 3′ untranslated regions (UTRs) of target mRNAs. Their biogenesis involves sequential processing by Drosha and Dicer enzymes to yield mature miRNAs. During infection, flaviviruses can alter host miRNA profiles to create a cellular environment favorable for viral survival. Some host miRNAs directly bind to conserved regions of the flavivirus genome, particularly the 3′ UTR, suppressing or enhancing viral replication. Additionally, flaviviruses interact with complex non-coding RNA networks, including long non-coding RNAs and circular RNA, which act as competing endogenous RNAs to regulate miRNA activity. Viral-derived RNAs such as subgenomic flaviviral RNAs and virus-derived small RNAs further modulate replication and pathogenesis. Computational studies have identified several human and mosquito miRNAs with potential antiviral activity, offering promising leads for therapeutic targeting of flavivirus infections. A list of miRNAs involved in host-pathogen interactions has been reported in Cai et al.

Beyond antigen and platform development, understanding the host’s immune response at a deeper level is vital. Cai et al.‘s analysis, focusing on how host microRNAs (miRNAs) change during flavivirus infection, shifts our attention to cellular interactions. Although not directly a vaccine approach, this research offers crucial insights into immune evasion mechanisms and cellular regulation. These insights can be exploited in immunization strategies by:

- Identifying targets for co-administration with vaccines to enhance the host environment and immune response.
- Discovering new antiviral drugs for prophylactic or therapeutic use to lower viral loads, improving vaccine efficacy.

## Vaccine development against flaviviruses

Several flaviviral vaccines have been approved worldwide to combat diseases caused by arthropod-borne viruses in the Flaviviridae family (Immunization, vaccines and biologicals who.int, 2025). For yellow fever, two widely used vaccines are YF-VAX (Sanofi Pasteur, USA) and Stamaril (Sanofi Pasteur, France), both based on the live-attenuated 17D strain and approved in the 1990s and early 2000s, respectively. Japanese encephalitis vaccines include Ixiaro (Valneva SE, Austria, approved in 2009), SA 14-14-2 (Chengdu Institute, China, approved in 1989), JEEV (Biological E Ltd, India, approved in 2013), and IMOJEV (Sanofi Pasteur, Thailand, approved in 2010). For dengue, Dengvaxia (Sanofi Pasteur, France) was the first approved vaccine in 2015, followed by QDENGA (Takeda, Japan) in 2022, which has demonstrated broader efficacy across serotypes. Tick-borne encephalitis vaccines include FSME-IMMUN (Pfizer, Austria, approved in 1976) and Encepur (GSK, Germany, approved in 1991). A notable advancement in alphavirus vaccine research is the FDA’s authorization of two CHIKV vaccines: IXCEHIQ, a single-dose live-attenuated formulation approved in November 2023 (FDA, 2023), and VIMKUNYA, a recombinant virus-like particle (VLP) vaccine formulated with aluminum hydroxide as an adjuvant, approved in February 2025 (Vimkunya, 2025).

## Guiding the immune response: focus on precise epitopes and T cells

The future of flavivirus vaccines depends heavily on precisely designed antigens, shifting from whole-virus or basic subunit approaches to carefully selected epitopes. A prominent theme is the focus on cellular immunity. While vaccines traditionally aim to elicit neutralizing antibodies, long-lasting protection and infected cell clearance rely on CD4^+^ and CD8^+^ T cells.

The contribution to this Research Topic by Eickhoff et al. exemplifies this shift by identifying immunodominant T cell epitopes from ZIKV that are unique and not cross-reactive with DENV. This provides essential elements for T cell-targeted vaccines that reduce the risk of enhancing DENV infection—an important safety concern in regions where multiple flaviviruses co-exist. Similarly, Shinian et al. also used of immunoinformatics to design a nanovaccine with a stable ferritin platform demonstrates a robust, computational method. This approach enables efficient screening and assembly of B-cell and T-cell epitopes into a highly organized, self-adjuvanted candidate that stimulates both immune pathways. This establishes a valuable precedent for rapidly developing multivalent flavivirus vaccines.

Despite progress in developing vaccines against various flaviviruses, dengue vaccine development deserves priority, as dengue poses the highest global burden among them, affecting over half of the world’s population with a rapidly rising incidence. Unlike other flaviviruses, dengue’s four serotypes cause ADE, in which partial immunity worsens subsequent infections, making it an urgent immunological and public health challenge. In this Research Topic, Parra-Gonzalez et al. have presented a comprehensive review on the safe and efficacious vaccine development against Dengue. This review highlights VLP vaccines as safe, non-replicating alternatives that mimic native dengue virus structure to elicit balanced neutralizing antibodies without ADE risk.

## Addressing cross-reactivity and the ADE issue

Design precision is especially crucial when tackling the major challenge in flavivirus vaccines: ADE. ADE occurs when pre-existing, non-neutralizing antibodies (from previous infections with related flaviviruses) facilitate viral entry into host cells, causing more severe disease. Weiss et al.‘s work with WNV illustrates how rational antigen modification can overcome this issue. By strategically mutating the conserved fusion loop (FL) in the E protein or using only E Domain III, they reduced serological cross-reactivity with other flaviviruses while maintaining protective immunity against WNV. This supports the strategy of altering or removing conserved epitopes to develop safer vaccines for regions with multiple circulating flaviviruses.

Parra-González’s review further advocates using Virus-Like Particles (VLPs) for DENV. VLPs, which cannot replicate, are inherently safer. Engineering these VLPs to exclude the prM protein—a component linked to ADE—provides a sophisticated, forward-looking solution. This ensures the antibodies produced are mainly neutralizing, reducing the risk of enhancement that has hindered previous DENV vaccine efforts. Advances in recombinant expression systems, mammalian, insect, yeast, and plant platforms, have enhanced VLP yield, stability, and scalability. Novel formulations, including prM-free designs and thermostabilized preparations, further improve safety and efficacy. The review also emphasizes integrating these scientific and logistical innovations to establish dengue VLP vaccines as viable next-generation tools for global dengue prevention.

WNV, which causes severe neurological disease in humans, does not have a licensed human vaccine. In this Research Topic, Weiß et al. presented an original work comparing the immunogenicity and cross-reactivity of three recombinant WNV E protein constructs: wild-type E (Ewt), fusion loop-mutated E (Equad), and domain III (EDIII) in mice. All antigens generated strong WNV-specific antibody responses, but Ewt produced the highest neutralizing titers and full protection against lethal infection. Equad and EDIII offered partial protection while significantly reducing cross-reactive antibody responses to other flaviviruses like ZIKV and Usutu viruses. The results emphasize the role of the fusion loop domain in antibody cross-reactivity and reveal a key trade-off between specificity and protective efficacy. Modified or truncated E antigens could improve the safety of WNV vaccines by reducing ADE risk, guiding the development of next-generation flavivirus vaccines.

ZIKV infection is linked to congenital brain abnormalities and Guillain-Barré syndrome and lacks a licensed vaccine. As noted, Eickhoff et al. have identified ZIKV-specific CD4^+^ and CD8^+^ T cell epitopes induced by natural infection using a combination of immunoinformatics and experimental validation. They have employed both overlapping peptide screening and MHC-binding prediction algorithms across six class I and nine class II HLA supertypes. IFN-γ ELISPOT assays using PBMCs from ZIKV-infected individuals revealed 31 MHC class I-restricted and 27 MHC class II-restricted immunogenic epitopes distributed across structural and non-structural proteins. These epitopes showed strong immunodominance, broad population coverage, and minimal cross-reactivity with dengue virus. The study demonstrates that computational prediction-based strategies outperform overlapping peptide methods in identifying relevant epitopes. Overall, these findings provide a valuable epitope repertoire for the rational design of T cell–targeted Zika vaccines that elicit broad, protective cellular immunity.

## Future directions

Broad strategies for vaccinating against flaviviruses are rapidly developing to address safety, effectiveness, and cross-protection issues caused by viruses like dengue, Zika, and West Nile. These include nucleic acid platforms such as mRNA and DNA vaccines that allow quick antigen production and strong immune responses, VLPs that simulate native viral structures without genetic material, and recombinant viral vectors that efficiently deliver flaviviral antigens. Structure-guided antigen design aims to expose neutralizing epitopes while reducing ADE, and multivalent or chimeric vaccines are being created to provide broad protection across flavivirus serotypes. Additionally, new delivery methods like microneedle patches and advanced adjuvants improve accessibility and immune response, while pan-flavivirus approaches targeting conserved non-structural proteins show potential for universal protection.

Moreover, international cooperation is crucial for enhancing global vaccine distribution by addressing logistical, financial, and equity issues. Key approaches involve ensuring fair access to prioritize vulnerable, low-income groups and creating global manufacturing centers to improve scalability and reduce costs. Additionally, coordinated efforts promote sharing of resources, expertise, and disease surveillance data, along with standardized production systems. This comprehensive strategy ensures effective funding, tackles public hesitancy, and ultimately expands the reach and effectiveness of immunization initiatives worldwide.

## Conclusion

In summary, the articles compiled in this Research Topic highlight significant advances in understanding flavivirus pathogenesis and vaccine development. The integration of novel vaccine platforms, structural insights, and computational tools offers promising avenues for achieving safe, broad-spectrum vaccines. Success will depend on precisely designed antigens that prioritize T-cell responses, along with advanced, safe platforms like VLPs and nanoparticles engineered to minimize adverse effects like ADE. A detailed understanding of host-virus molecular interactions must inform ongoing research. Collectively, these studies mark significant progress toward better, broadly protective flavivirus vaccines. Continued collaborative research efforts, particularly between academia and industry, will be essential to translate these findings into effective and affordable vaccines, ultimately strengthening global preparedness against flaviviral infections. Overall, flavivirus vaccines play a pivotal role in improving global public health by preventing disease, controlling outbreaks, and reducing the socioeconomic burden of these infections.
